# Host Transcript Accumulation during Lytic KSHV Infection Reveals Several Classes of Host Responses

**DOI:** 10.1371/journal.pone.0000811

**Published:** 2007-08-29

**Authors:** Sanjay Chandriani, Don Ganem

**Affiliations:** Howard Hughes Medical Institute, George Williams Hooper Foundation, University of California at San Francisco, San Francisco, California, United States of America; University of Hong Kong, China

## Abstract

Lytic infection by Kaposi's sarcoma-associated herpesvirus (KSHV) is associated with an extensive shutoff of host gene expression, mediated chiefly by accelerated mRNA turnover due to expression of the viral SOX protein. We have previously identified a small number of host mRNAs that can escape SOX-mediated degradation. Here we present a detailed, transcriptome-wide analysis of host shutoff, with careful microarray normalization to allow rigorous determination of the magnitude and extent of transcript loss. We find that the extent of transcript reduction represents a continuum of susceptibilities of transcripts to virus-mediated shutoff. Our results affirm that the levels of over 75% of host transcripts are substantially reduced during lytic infection, but also show that another ∼20% of cellular mRNAs declines only slightly (less than 2-fold) during the course of infection. Approximately 2% of examined cellular genes are strongly upregulated during lytic infection, most likely due to transcriptional induction of mRNAs that display intrinsic SOX-resistance.

## Introduction

Kaposi's sarcoma-associated herpesvirus (KSHV) is a lymphotropic (gamma-2) herpesvirus that is etiologically linked to Kaposi's sarcoma (KS) as well as to several lymphoproliferative syndromes [1]–[5]. Like all herpesviruses, KSHV displays two alternative genetic programs, latency and lytic replication. In latency, viral gene expression is restricted to a handful of genes [6]–[8], and the viral genome is maintained in the nucleus as a low copy-number episome [9]; host gene expression continues unabated, and no viral progeny are produced. Latent infection is the default program for viral infection in culture [10]. However, latently infected cells retain the full viral genome and, under the appropriate conditions, can be induced to enter the lytic cycle [11], [12]. In this state, the majority of viral genes are expressed according to a temporally regulated program, with immediate-early (IE) genes expressed first, followed by delayed-early (DE) genes [13]. IE proteins generally serve as regulators of the subsequent classes; the key IE protein is RTA (replication and transcription activator), a transcription factor that is responsible for the switch from latent to lytic replication [11], [14], [15]. Many DE genes are directly upregulated by RTA, which can activate transcription by direct DNA binding or by recruitment to other promoter sites through binding to cellular transcription factors (RBP-Jk, C/EBPα, and others)[16]–[21]. DE gene expression triggers lytic DNA replication, following which late (L) genes, predominantly encoding virion structural proteins, are expressed. During the late phase of lytic replcation, infectious viral progeny are assembled and released in large numbers.

Although most tumor cells of KS are latently infected, the lytic cycle is thought to play an important role in KS tumorigenesis, since ganciclovir treatment, which specifically blocks lytic replication, leads to a prompt and significant reduction in KS development even after many years of KSHV infection [22]. This suggests that the continuous operation of the lytic cycle in some fraction of the infected cells is necessary to sustain KS tumorigenesis. How the lytic cycle contributes to KS development has been a matter of debate [13]. One model posits that growth and angiogenic factors released from lytically infected cells may influence tumor progression in a paracrine fashion. Many such factors are encoded by DE viral genes that have been identified, which include virally encoded cytokines and chemokines (e.g. v-GPCR, v-CCL1, v-CCL2, v-CCL3, v-IL6) (review,[23]). An important remaining question has been whether host genes encoding such factors can be induced by lytic infection. For example, the G-protein coupled receptor encoded by the DE gene ORF74 [24] can induce VEGF production when expressed in uninfected cells in culture [25], and can result in angiogenesis in surrounding tissues when expressed as a murine transgene [26]. However, the relevance of these in vitro observations to in vivo infection depends on whether the indicated host genes can be expressed in the environment of lytic infection. Since some (but not all) herpesviruses induce a shutoff of host gene expression during their lytic cycle, investigation of the ability of KSHV-infected cells to support host gene expression is important to evaluating the potential contributions of host gene products to paracrine signaling.

For this reason, our laboratory recently examined whether host gene expression is affected during the KSHV lytic cycle. Our results showed that KSHV infection leads to a rapid and extensive shutoff of host protein synthesis, as judged by pulse labeling with ^35^S-methionine [27]. Further studies revealed that this is due to the action of a single viral gene, now termed SOX (shut-off and exonuclease), which leads to a large-scale degradation of host mRNAs [27]. Subsequently, we examined the profiles of host mRNAs in infected cells to see if any host transcripts could escape SOX-mediated decay [28]. In those studies, microarray data were normalized with the common assumption that the levels of relatively few transcripts change from condition to condition. Therefore, we were able to identify only the most highly expressed escapees that appear to be resistant to the inhibitory effects of SOX. Here, we re-examine host mRNA expression in lytically infected cells using a normalization algorithm that can reveal global gene expression changes if they occur; this allows a more comprehensive view of the magnitude and extent of host mRNA transcript loss. Our results show that circa 75% of transcripts are massively downregulated during lytic replication, with fewer than 2% undergoing active upregulation during the lytic cycle. Moreover, we identify a previously unrecognized class of mRNA whose levels decline only modestly (less than 2-fold) during lytic growth. This class is not small, representing approximately 20% of transcripts studied. Our results support the view that there is a gradient of susceptibility to SOX regulation among host transcripts, but affirm that *de novo* upregulation of host mRNAs in response to infection is limited to a very small subset of the transcriptome; this subset includes several paracrine signaling molecules, though VEGF is not prominent among them.

## Materials and Methods

### Tissue culture, virus preparation and infection

TIME cells were cultured as previously described[29]. Briefly, cells were expanded in EGM™-2 MV (Clonetics). KSHV virus stocks were prepared from BCBL-1 cells as previously described [10]. Virus titers were empirically determined by serial dilution and infection of TIME cells. For expression profiling experiments, cells were infected with KSHV (or mock infected) under conditions that yielded approximately 95% latently infected cells 48 hours post infection as judged by staining for the latency-associated nuclear antigen (LANA) (data not shown). After 6 hours of exposure to the KSHV inoculum, cells were washed with PBS and subjected to infection by Ad-RTA (adenovirus harboring the RTA cDNA) under conditions that yielded nearly 80% of KSHV-infected cells undergoing lytic cycle 48 hours later as judged by staining for the lytic marker, Orf59. Time = 0hr refers to the time at which Ad-RTA was applied to cells.

### RNA preparation, labeling, and microarray hybridization

Total RNA was prepared from cells at times and conditions indicated in the text using the RNeasy Mini Kit (Qiagen) according to the manufacturer's protocol. Reference RNA was a mixture of RNAs purified from dividing untransduced human foreskin fibroblast, TIME cells and human umbilical vein endothelial cells (HUVEC). The integrity of the purified RNA's was analyzed using the 2100 Bioanalyzer (Agilent). RNAs were quantified using the ND1000 spectrophotometer (Nanodrop). The Low RNA Input Linear Amplification Kit (Agilent) was used according to the manufacturer's protocol to generate labeled cRNA from 240ng of total RNA. Experimental samples (labeled with Cy5) and reference samples (labeled with Cy3) were competitively hybridized to Whole Human Genome Oligo Microarrays (Agilent) according to the manufacturer's protocol. These microarrays have 41,000 probes that map to 20,087 unique unigene cluster IDs (Unigene Build #188). Cyanine 3-CTP and Cyanine 5-CTP were obtained from Perkin Elmer. Hybridizations and washes were performed according to the manufacturer's protocol. Washed arrays were scanned using the 48-slide DNA Microarray Scanner (Agilent) and feature intensities extracted using Feature Extraction Software version 8.5 (Agilent). All raw data were stored in the MIAME (minimum information about a microarray experiment)-compliant Princeton University MicroArray Database (PUMAdb) and can be accessed at http://puma.princeton.edu/. Fully normalized and processed data can be accessed at http://www.ucsf.edu/micro/faculty/ganem_folder/data/chandriani/shutoff/KSHV_lytic.html.

### Microarray Data Analysis

Features on the array that were flagged as “Population outlier” or “Non-uniformity outlier” were removed from the data and not considered further. Both green and red channel intensities were required to be well above background. Furthermore, only features for which the green channel median intensity was greater than 85 and the red channel median intensity was greater than 110 were considered for further analysis. LOWESS normalized log_2_-ratios of features that passed these spot quality filters were downloaded from PUMAdb. For the second normalization step, we utilized signals from probes against the spiked transcripts that passed the following spot quality filters: were not feature or background “non-uniformity outliers”, were not saturated and were “well above background.” The LOWESS normalized log_2_-ratios for these features were averaged for each array. This average value constitutes the second normalization factor linearly applied to the remaining probes on the array.

The completely normalized data were then subject to zeroing and other filters. For a given probe in the time course series, the average log_2_-ratio across the two zero hour samples (mock KSHV) was subtracted from every log_2_-ratio expression value across the lytic replication time course experiments. In the time course data, probes that displayed at least a 1.86 fold change (from the 0 hr, mock KSHV) in at least two time points and had greater than 75% present data were considered responsive. As explained in the text, one unique cluster of 66 probes whose changes could not be validated by quantitative RT-PCR was removed for further consideration (the complete list of these probes is available in supplementary Table S1). These filters yielded a group of 10,006 probes.

Because we did not expect global changes in host gene expression upon over expression of vGPCR, RTA or EGFP, we only performed LOWESS normalization on these 9 arrays. A similar zeroing was conducted in this follow-up study except the three mock infected samples served as the zeroes. Probes were filtered for 80% present data and then filtered for at least two arrays displaying a 1.86 fold change over the average of the mock infected samples. These filters yielded a group of 2,392 probes.

Hierarchical clustering of data was performed with Cluster 3.0 [30]. Clustered data are displayed at a heat map using Java TreeView [31].

### ARE enrichment

To identify ARE-containing transcripts, 3′UTRs for all available transcripts represented on the microarray were downloaded from the BioMart Project (http://www.biomart.org/biomart/martview). 3′ UTR sequences were available for 15,372 genes represented on the array. This information was used to query genes that contain the octomer ARE sequence, UAUUUAWW (W = A or U), at least twice in their 3′ UTRs [32]. The significance of enrichment of ARE-containing transcripts among the escapees over the background representation in all the genes on the array was calculated by assuming a hypergeometric distribution of ARE elements in 3′ UTRs. Analysis was performed in Matlab.

### qRT-PCR

Quantitative RT-PCR was performed to corroborate microarray results for several genes. cDNA was generated using random primers with 2ug of total RNA using Superscript III reverse transcriptase according to the manufacturer's protocol (Invitrogen). A small fraction (1/50) of the reaction serves as the template for real-time PCR using specific primer/probe sets for each gene from Applied Biosystems. Quantitative PCR was performed using the 7300 Real Time PCR system (Applied Biosystems). Because host shutoff would likely affect commonly used transcripts for normalization, we also measured rRNA levels by RT-PCR and used this data for normalization of the qPCR results. The ddCt data (from qPCR) can be interpreted as normalized expression levels in log_2_ space. This data is therefore easily compared to the microarray data, which is presented as log_2_-ratios.

## Results

### Experimental design

Many different cell types comprise the hallmark angioproliferative lesions in KS; however, only the spindle cells, which are of endothelial origin, are infected with KSHV [33]–[35]. Therefore, we designed our experiment to capture transcriptome-wide changes in a microvascular endothelial cell line called TIME cells [29]. Time cells were first treated with mock or KSHV inoculum for 6 hours. Inoculum was removed, cells were washed and then subjected to infection by an adenovirus construct encoding KSHV RTA (Ad-RTA). Total RNA was harvested at 0, 1.5, 3, 13, 24, 37 and 48 hours following Ad-RTA infection. Duplicate (for the mock KSHV infected cells) or triplicate (for the KSHV infected cells) zero time points were collected whereas single samples were collected for the ensuing time points. To evaluate host gene expression changes in response to isolated expression of either vGPCR or RTA, naïve TIME cells were infected in duplicate with adenovirus constructs encoding for vGPCR, RTA, EGFP or mock infected (in triplicate). Total RNA was harvested 48 hours post infection. Genome wide expression studies were performed using Whole Human Genome Oligo Microarrays from Agilent (see materials and methods).

### Microarray normalization

Commonly, microarray data normalization methods assume that relatively few transcripts change from sample to sample [36]. Therefore, most normalization algorithms of two-color microarray data effectively move the distribution of log(R/G) of all the features on an array to zero [37]. Even the more sophisticated normalization algorithms (such as LOWESS) that apply a non-linear correction to the features to accommodate intensity dependent biases also have an underlying assumption that relatively few transcripts change from sample to sample. When global changes in gene expression are predicted, external normalization controls can be used to better assess these changes[36]. We used a modification of a method described by van de Peppel et al. [36] to assess transcript changes during lytic replication; in this method, an equal amount of a mixture of 10 different in-vitro synthesized transcripts are added to an equal amount of total RNA of each sample. The Agilent microarray harbors probes that specifically recognize these spiked transcripts and can serve as critical internal standards for normalization. In so doing, the underlying assumption is that total RNA levels do not substantially change from sample to sample. (We experimentally validated this assumption by comparing total RNA yields from cells that are undergoing lytic replication and those which are not; data not shown). We used these exogenous transcripts to normalize the endogenous transcripts in a two-step normalization protocol. In the first normalization step, we utilized a LOWESS algorithm based on all the probes of the array. In the second step, we performed a linear normalization based only on the intensities of the probes for the spiked transcripts. We compared the microarray data normalized under the assumption that no global effects are taking place (LOWESS) versus data normalized without that assumption (two-step normalization) and observed starkly different expression profiles (supplementary Figure S1). To empirically validate the normalization protocol that better approximates changes in transcript levels, we used quantitative RT-PCR as an independent method to measure transcript level changes of more than 30 different mRNAs (see below). The subsequent RT-PCR data affirm (see below) that the microarray data normalization using spike-in controls more accurately reflects underlying changes to cellular transcripts during lytic replication.

### Transcript accumulation during KSHV lytic replication

**Figure 1:**
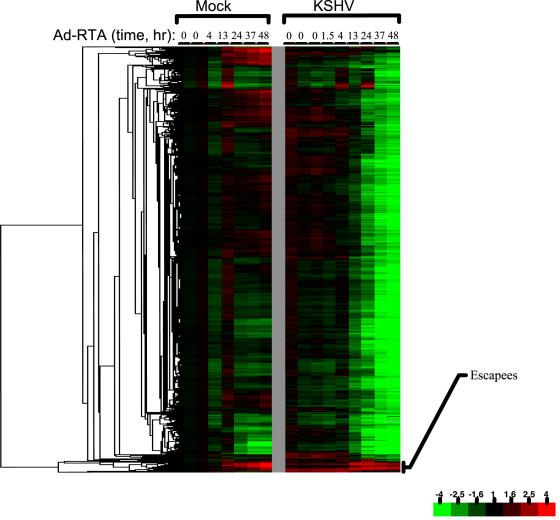
Host (TIME cells) mRNA expression data during KSHV lytic infection. Clustered microarray data are displayed for 10,006 probes exhibiting a 1.9 fold change (relative to the average of the 0hr timepoints of the mock KSHV/Ad-RTA infected cells) in at least two time points. Detailed description of data filters is available in the Materials and Methods. The color bar describes the fold changes with respect to mock infected cells at time zero.

**Figure 2:**
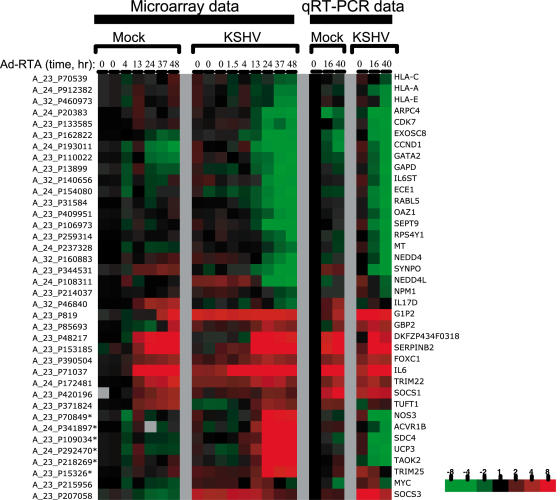
Validation of microarray results by quantitative RT-PCR. Normalized log_2_-ratios of the microarray data and the average ddCt qPCR values of select genes are directly compared in colorimetric form. Probe names are given for the microarray data and the corresponding short gene names are given along side the qRT-PCR data. Asterisk (*) indicates microarray data that were not confirmed by qRT-PCR. As noted in the Results section, these probes are part of the cluster of microarray probes that was removed from subsequent analyses. The color bar refers to the fold changes with respect to mock infected cells at time zero.

**Figure 3:**
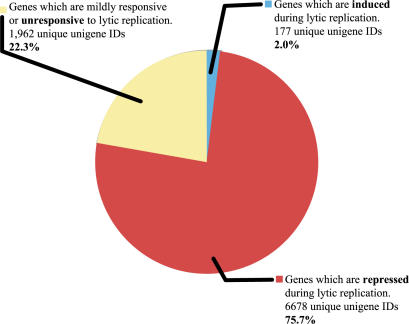
Summary of changes in transcript levels during KSHV lytic infection. Pie chart represents the total number (8,817) of unique unigene cluster IDs that pass microarray spot quality filters in 75% of arrays. The three sectors represent genes that display changes in the indicated fashion. The filters used to define the induced and repressed are the same as in Figure 1. The unresponsive sector represents genes that did not pass the fold filter, but did pass the spot quality filters.

To display transcripts that show even mild changes over the course of infection, a non-stringent fold filter (at least a 1.9-fold change in at least 2 time points) was applied to the expression data (Figure 1). Immediately, it is apparent that the levels of the vast preponderance of RNA transcripts decline substantially and progressively with infection, as predicted by our earlier studies of SOX-transfected cells. We sampled 16 representative examples of these RNAs by quantitative RT-PCR to independently confirm the downregulation seen on the array; the direction of transcript level change (i.e. down) was confirmed in every case (Figure 2). More importantly, as a validation of the two-step normalization, the magnitude of the changes as assessed by quantitative RT-PCR agreed very closely with the magnitude of changes suggested by the microarray data. Only a small number of mRNAs actually increase in abundance during infection; many, but by no means all, of these appear to be inducible by Ad-RTA alone (see below). Figure 3 displays a schematic depiction of the array results, grouped according to the nature and magnitude of transcript level changes occurring during lytic growth. Over 75% of the host mRNAs whose signals met our stringent spot quality filters were strongly downregulated during lytic growth. To our surprise, another 22% of host transcripts declined only modestly in abundance during infection (mRNAs were assigned to this class if their levels declined by less than 1.9 fold during infection and are not displayed in the heat map of Figure 1). Again, quantitative RT-PCR analysis of 5 out of 5 randomly chosen members of this class also affirmed the array results (Figure 2). We think it likely that most of these transcripts are only weakly responsive to SOX-induced turnover; however, we cannot exclude that some of them are RNAs whose SOX-mediated turnover is balanced by transcriptional upregulation. Another small subset of mRNAs may be erroneously included in this class. We believe that some transcripts are so severely downregulated that the late time points fail to pass the spot quality filter of having spot intensity well above background; such RNAs would be assigned to this class by the above analysis. Overall, these results indicate that circa 98% of host transcripts remain at or below pre-infection levels during KSHV lytic growth.

### mRNAs that escape KSHV mediated host shutoff

**Figure 4:**
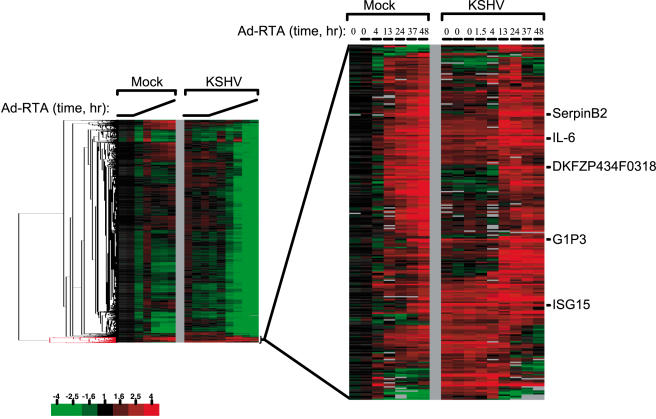
mRNAs that escape KSHV mediated host shutoff. An expanded view of one cluster of mRNAs that exhibit elevated levels during lytic replication is displayed. Probes that map to select mRNAs are annotated. The color bar describes the fold changes with respect to mock infected cells at time zero.

Approximately 2% of assayed host mRNAs increased in abundance during infection. In our initial analyses, several small clusters of seemingly upregulated transcripts were observed (see supplemental Figure S2). However, for one of these clusters quantitative RT-PCR measurements failed to confirm the upregulation in 6 out of 6 cases (Figure 2). The reasons for this remain obscure, however, we suspect that some viral transcripts that are highly expressed during lytic replication may cross-hybridize to these microarray probes. Therefore, the 66 probes, which map to 54 genes, in this entire cluster were excluded from the summary analyses of Figure 3. Other upregulated mRNAs outside of this anomalous cluster were readily confirmed by qRT-PCR (9 out of 9 representative samples) (Figure 2). Figure 4 shows the heat map of upregulated mRNAs in expanded form. As expected, several of these transcripts correspond to genes whose upregulation we had earlier observed by qualitative array analysis and whose upregulation at the protein level was confirmed by immunoassay or immunoblotting (e.g. IL-6) [28]. However, the present analysis greatly expands the list of identified upregulated RNAs, from 9 to 177. We attribute this to more accurate quantitation and normalization of transcript abundances, owing to the use of spiked transcript controls, as well as the use of larger arrays.

A full accounting of these upregulated transcripts is available in supplementary data (supplementary Table S2). While we do not yet understand the biology of all these gene products, there are tantalizing clusters of mRNAs with related functions. First, this endothelial cell line produces a number of proteins with activities in angiogenesis and vascular cell biology, including angiopoietin 2, angiopoietin-like polypeptide, ephrins A1 and B2, endothelin convertase and serpin B2 (plasminogen activator inhibitor 2). Second, several interferon-inducible transcripts accumulate (e.g. IFI 6-16, IFI 15, IFI 27, and GBP2), though the full complement of interferon-responsive mRNAs is not detectable. (SOX may degrade many of the latter.) In addition, however, we and others have recently found that KSHV encodes several lytic functions that blockade IFN signaling (Bisson, Page and Ganem, manuscript in preparation) [38]–[42]. It therefore remains possible that the small set of upregulated transcripts labeled as IFN-responsive are in fact being upregulated by other, as yet unknown, cellular pathways. The arrays also reveal ample evidence of cytokine dysregulation, including the profound induction of IL6 and CXCL2. The upregulation of IL6 (and the production of v-IL6) may explain another observation, namely, the upregulation of the counter-regulatory molecule SOCS-1 (suppressor of cytokine signaling), which acts to impair STAT 3 function downstream of the IL6 receptor [43]; however, many other explanations for SOCS1 upregulation are also possible. In addition, many molecules active in other cellular signal transduction pathways are upregulated, including many dual-specificity protein phosphatases, several orphan GPCRs, several ubiquitin ligases and components of the Notch signaling pathway (e.g. jagged 1, a ligand for Notch). Finally, numerous transcription factors are upregulated (e.g. FOXC1, ATF3, MADS-box TF1, HoxA5), but the significance of this remains to be established, since many of their induced transcripts will likely be subject to SOX-mediated decay.

### Enrichment of transcripts harboring ARE's among escapees

It is likely that there are multiple mechanisms by which transcripts escape SOX-mediated degradation. We have earlier shown that cis-acting sequences in transcripts play an important role in making transcripts refractory to SOX-mediated degradation [28]. Accordingly, we were interested to know if any cis-elements with known effects on RNA stability were over-represented in escapees of SOX-mediated decay. One well-known class of such elements are the so-called AU-rich elements (AREs). AREs bind multiple cellular factors, some of which stabilize and others of which destabilize the RNA [44]. In the ground state, the net result of this is destabilization of the transcript. However, certain stimuli (notably p38 activation) can inactivate one or more of the destabilizing proteins (e.g. tristetraprolin or TTP), resulting in net stabilization of ARE-containing mRNAs. Interestingly, KSHV encodes a protein (Kaposin B) that upregulates the p38 pathway and stabilizes ARE transcripts, and this protein is highly expressed during lytic replication [45]. Accordingly, we examined whether AREs might be enriched among the class of transcripts that escape shutoff. Indeed, when we examined the 3′ UTR sequences of the escapees, we found that this group of transcripts was enriched by 50% for transcripts that had two or more AREs in their 3′ UTRs (Table 1). Assuming a hypergeometric distribution, this enrichment is statistically significant (p-value = 1.0×10^−3^). However, we note that (i) many escapees lack AREs, and (ii) that many RNAs that are downregulated by SOX harbor AREs. Indeed, in one prominent SOX-resistant mRNA (IL6) that contains an ARE, mutation of the ARE does not restore SOX-susceptibility (Britt Glaunsinger, personal communication). Taken together, these data suggest that AREs cannot be the sole determinant of SOX resistance; multiple factors are likely at work.

**Table 1:** 

	Two or more ARE's in 3'UTR
Background	3287/15372 (21%)
Escapees	50/155 (32%)
Enrichment p-value	1.0×10^−3^

### Host gene expression changes induced by GPCR, RTA

**Figure 5:**
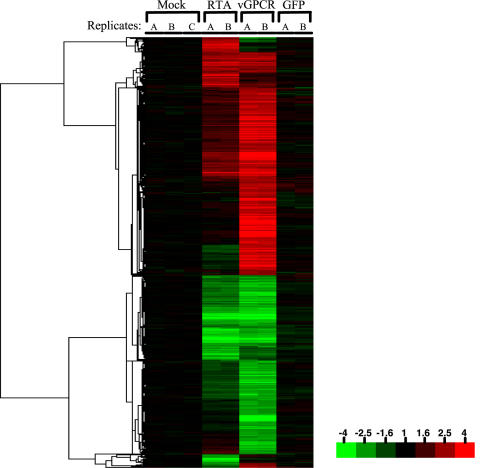
Host mRNA expression data upon expression RTA, vGPCR and GFP. RTA, vGPCR or GFP were ectopically expressed in TIME cells. 48hrs after adenoviral delivery of these cDNAs, samples were harvested and gene expression analysis was performed. Clustered microarray data are displayed for 2,392 probes exhibiting a 1.9 fold change (relative to the average of the three mock infected samples) in at least two arrays. Detailed description of the data filters is available in the Methods.

**Figure 6:**
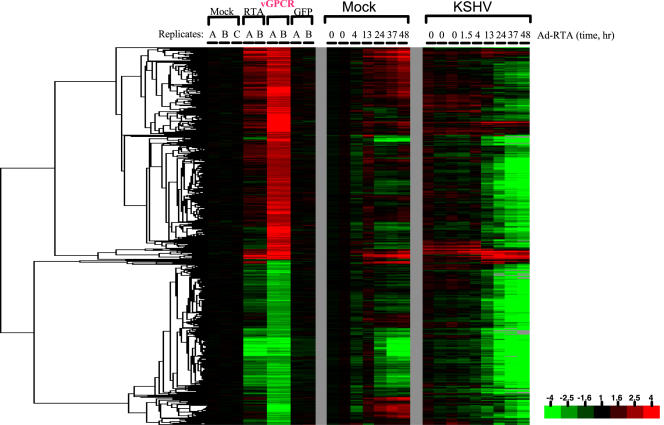
vGPCR responsive transcripts during lytic replication. Microarray data in figures 1 and 3 were linked. Clustered data are displayed for 1,902 probes exhibiting at least a 1.9 fold change (relative to the average of the three mock infected samples) in both vGPCR arrays. Detailed description of data filters is available in the Methods.

What is the origin of the upregulation of these 177 mRNAs? Two viral gene products that have been much discussed in these contexts are RTA and the viral GPCR (orf74). As shown in Figure 5, when each is expressed individually in TIME cells by an adenovirus vector, large numbers of host transcripts are induced. The set of upregulated genes induced by each regulator is distinct but overlapping. Figure 6 shows that when the set of GPCR-upregulated genes is considered, the vast majority are downregulated during lytic infection; only approximately 5% are upregulated during lytic growth. This result affirms the need for caution in extrapolating from results with GPCR-transfected cells to the circumstance of authentic infection.

**Figure 7:**
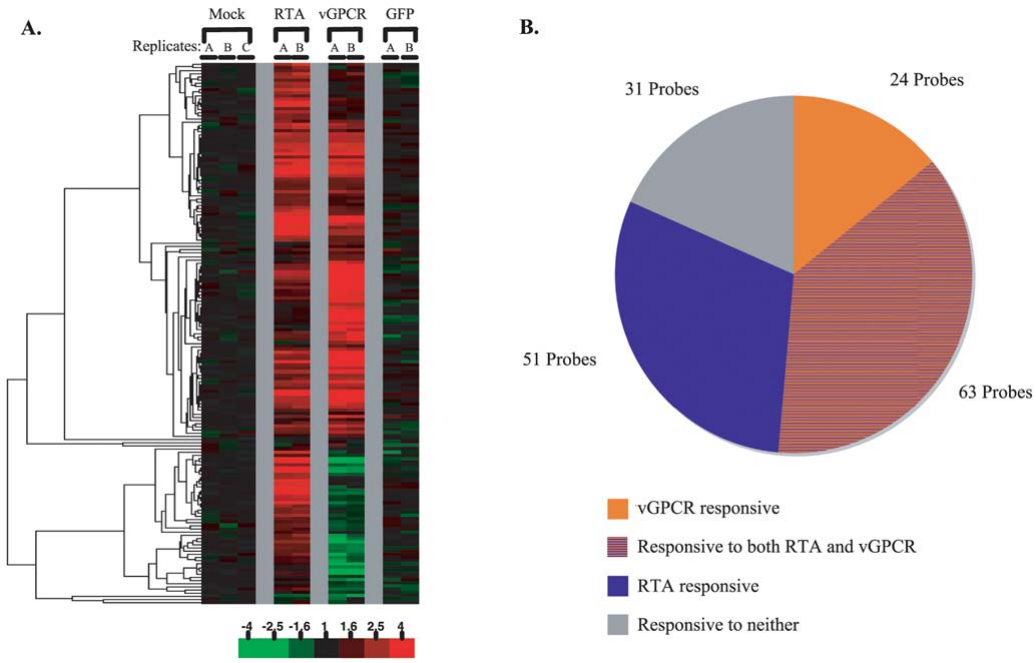
Response of escapees to isolated expression of RTA, vGPCR or GFP. A. Expression data for mRNAs that escape KSHV mediated host shutoff (Fig. 1) are selected (169 probes) from the dataset presented in Fig. 3. B. These data are summarized in a pie chart that depicts the influence of vGPCR and RTA on lytic escapee transcripts.

Figure 7 shows another way to depict the potential contributions of GPCR and RTA to those few genes that are upregulated during lytic growth. Of the 169 probes analyzed, only 14% were uniquely induced by vGPCR expression; fully 30% were induced solely by RTA expression, and another 37% can be upregulated by either viral protein. Of course, this correlative analysis does not prove that these host genes are being upregulated by *these* viral proteins in vivo; this analysis is presented to outline the maximal potential contributions of each regulator to the observed patterns). Notably, nearly 18% of upregulated genes could be induced by neither viral factor, suggesting that other viral regulators likely also play important roles–candidates for these include the K1, K8, K14, MTA (orf 57) and K15 proteins. Dissecting the relative contributions of each of these factors to the overall pattern of host mRNA accumulation will likely require genetic and functional genomic analyses of each coding region, carried out in the context of lytic KSHV replication.

## Discussion

These studies represent a comprehensive examination of the host endothelial transcriptome during lytic infection by KSHV, using methods that allow quantitative assessment of the magnitude and scope of mRNA abundance changes as infection progresses. The data reveal that while a large majority of host transcripts (∼75%) succumb strongly to SOX-mediated degradation, a sizeable minority of transcripts (∼20%) is only mildly affected by SOX. Furthermore, a small subset of transcripts (∼2%) not only escapes host shutoff, but is in fact more highly expressed. We note here that although we have chosen (for clarity and simplicity) to categorize the responses to lytic infection as falling into 3 discrete classes, there is in fact a continuum of changes in host transcript levels ranging from strong induction to reduction below detection limits.

What accounts for the variable responses of host transcripts to the lytic environment? At present, we do not have definitive answers to this question, but we can point out several possibilities. First, some transcripts may bear cis-acting sequences that confer resistance to SOX-mediated degradation. We have previously described one such sequence in the 3′ UTR of IL-6 mRNA, an RNA that strongly accumulates as lytic infection progresses. However, these sequences are not conserved in other RNAs whose abundance increases during infection, suggesting that additional cis-acting elements may exist that contribute to escape from SOX-mediated turnover. Our analysis shows that ARE elements are enriched in RNAs that escape degradation during infection. This is interesting since KSHV encodes a protein, kaposin B, that is strongly upregulated during lytic growth and that stabilizes ARE-containing messages via activation of the p38/MK2 pathway [45]. However, this appealing model is complicated by the fact that many RNAs that contain AREs are nevertheless degraded during infection, indicating that other effects can override ARE-mediated stabilization. How such effects might operate is unknown. Stimulated by the observation that AREs are enriched in 3′ UTRs of transcripts that escape degradation, we searched for other elements that are conserved and enriched among this group of transcripts. Using MEME (Multiple Em for Motif Elicitation [46]), several conserved elements were detected in these 3′ UTRs; however, none of these was enriched among the escapees when compared to the background representation in the 3′ UTRs of all the genes on the array (data not shown). This finding does not exclude that possibility that cis-acting elements are functional in restraining degradation since elements may be in other parts of the transcript or are in a form that the MEME algorithm could not detect.

Another possibility is that some cis elements can direct mRNAs to areas of the cytoplasm where SOX-mediated decay is more active; variations in the efficiency of such targeting could also lead to variations in transcript accumulation during infection. The idea that cytoplasmic compartmentation of transcripts plays a role in accessibility to the degradative machinery could also provide an attractive explanation for why viral mRNAs are not degraded during lytic replication. Finally, we note that even without regional or other variations in RNA turnover efficiency, other mechanisms can produce variation in the net accumulation of transcripts. Most simply, strongly enhanced transcription of some mRNAs could lead to their net accumulation by simply outrunning the degradative rate. Of course, none of these mechanisms is mutually exclusive, and the complex phenotype observed in vivo could well be due to contributions from all of these mechanisms. Clearly, much remains to be learned about the regulation of RNA stability during lytic KSHV growth. Resolution of these issues can be expected to inform our understanding of both KSHV pathogenesis and the control of host mRNA turnover more generally.

## Supporting Information

Figure S1 Comparison of microarray data normalized using LOWESS or two step using spiked-in transcripts) algorithm. Microarray data for experiment in Figure 1 were normalized using two different algorithms. These data were then linked together. The probes in Figure 1 were extracted, clustered and displayed as this heat map. The color bar describes the fold changes with respect to mock infected cells at time zero.
(0.85 MB DOC)

Figure S2 Microarray data including small cluster of probes which was removed from analysis. Clustered microarray data are displayed for probes exhibiting a 1.9 fold change (relative to the average of the 0hr timepoints of the mock KSHV/Ad-RTA infected cells) in at least two time points. These data include the small group of probes that were discarded from all analysis after qRT-PCR failed to validate this group. The color bar describes the fold changes with respect to mock infected cells at time zero.
(0.55 MB DOC)

Table S1 Pre-clustering file of microarray data presented in Figure 1.
(2.32 MB TDS)

Table S2 Pre-clustering file of microarray data of the escapees.
(0.05 MB TDS)
